# Uses, abundance perception, and potential geographical distribution of *Smilax aristolochiifolia* Mill (SMILACACEAE) on the Totonacapan Region of Puebla, Mexico

**DOI:** 10.1186/s13002-021-00477-6

**Published:** 2021-08-23

**Authors:** José Espinoza-Pérez, César Reyes, Jesús Hernández-Ruíz, Maximino Díaz-Bautista, Francisco Ramos-López, Abel Espinoza-Gómez, Oscar Pérez-García

**Affiliations:** 1 Posgrado en el Departamento de Agricultura, Sociedad y Ambiente, El Colegio de la Frontera Sur-Unidad San Cristóbal, San Cristóbal, Chiapas Mexico; 2 Dirección de Investigación y Posgrado, Universidad Intercultural del Estado de Puebla, Calle Principal a Lipuntahuaca, Lipuntahuaca, Huehuetla, Puebla Mexico; 3 División de Ciencias de la Vida, Universidad de Guanajuato, Carretera Irapuato-Silao, km 9, Ex Hacienda El Copal, Irapuato, Guanajuato Mexico; 4 Dirección de Ciencias Naturales, Universidad Intercultural del Estado de Puebla, Calle Principal a Lipuntahuaca, Lipuntahuaca, Huehuetla, Puebla Mexico

**Keywords:** Agroforestry systems, Potential distribution, Traditional knowledge, Use value, Wild edible plants

## Abstract

**Background:**

In some regions of Mexico, edible wild plants have been displaced or eliminated from the traditional food systems, mainly by changes in land use, booming monoculture, herbicide use, and by changes among the new generations in the traditional foods and diets of indigenous populations. In the Totonacapan region of Puebla, the gradual change from the traditional acahual plantation to coffee-type agroecosystems has provoked the displacement of old-growth forests and the eradication of wild plants since 1970. One of the wild species which has been used in traditional medicine and food recipes by the Totonac culture is *Smilax aristolochiifolia* (SMILACACEAE), known as “kgentsililh”. This species forms part of traditional Totonac recipes, in which the tender stems are still used in local medicine to treat menstrual pain, deal with dysentery, and prevent hair loss. According to the Maxent® Program, there are still potential areas with habitats suitable to promote its conservation in the Poblano Totonacapan.

**Methods:**

We conducted 260 interviews with people in 13 locations in the northern Sierra of the State of Puebla. Variables taken into account in the interview related to the consumption frequency of the species, its abundance and distribution perception, reasons or arguments given by the Totonac indigenous population about the decreased presence of specimens of *S. aristolochiifolia*, its dates of collection, and the cutting prices of kgentsililh at the community level and in local markets. The relative abundance of *S. aristolochiifolia* was determined through 22 samples in 2ts of 600 m^2^. Later, its potential distribution in the state of Puebla was estimated using the Maxent® Program Ver. 3.3.3.

**Results:**

Of the 260 Totonac families interviewed, 31% had stopped consuming kgentsililh. The residents reported that in the last 50 years the populations of this plant had diminished in the northern Sierra of the State of Puebla, mainly due to changes in land use, herbicide application, over-collection, and urban growth. In traditional medicine, the stem sap of *S. aristolochiifolia* is currently employed to help treat baldness, and the “tuberous root” or plant rhizome is used to prepare a tea infusion to treat dysentery. The cost of plant guides varies from 10.00 to 40.00 Mexican pesos for one bunch (around 0.5 to 2.00 US dollars), and every bundle consists of between 7 to 10 cuttings from 0.4 to 0.5 m long. From our 22 quadrats of sampling and collection of *S. aristolochiifolia*, we were able to recognize a total of 32 specimens. There is a considerable abundance of kgentsililh in acahual plantations and old-growth forests (evergreen lowland and mid-elevation perennial forest) concerning the coffee-type plantations and milpas. According to our analysis using the Maxent Program®, eight physical and climatic variables have a direct relationship to the potential distribution of the species.

**Conclusions:**

*Smilax aristolochiifolia* is still a plant of socioeconomic importance, mainly because of its food value and its use in traditional medicine by indigenous families in Poblano Totonacapan. It is evident that the villagers perceive that in the last 50 years the species has decreased its population mainly due to land-use change, the application of herbicides to the different family production units, and climate change. At the moment, there is no knowledge about the methods of propagation of the species, and therefore there is no intention on the part of the population to conserve the species. However, it would be of great importance to generate a biocultural conservation strategy and take advantage of the results obtained from the potential geographic distribution area, since according to the Maxent® Program, there are still potential areas with habitat suitable to promote conservation in Poblano Totonacapan.

**Supplementary Information:**

The online version contains supplementary material available at 10.1186/s13002-021-00477-6.

## Background

In Mexico, according to the ethnobotanical database of Mexican useful plants (BADEPLAM) of the Botanical Garden of the Biology Institute of the National Autonomous University of Mexico (UNAM), there are 7647 reported plant species considered to be useful species, among them plants that are edible, medicinal or ornamental, or that have more than 20 other uses. Of this group, 2168 are edible and may be encountered as wild, domesticated, weeds, or ruderal plants [1] with many different ways of management [2, 3]. Most of the wild edible plants collected here represent around 15% of the diet ingredients of the indigenous rural Mexican population [4], although between 65 and 118 plant species have been domesticated [5, 6]. However, several significant factors are creating a risk of loss of edible wild plants, such as changes in land use [7], deforestation of jungles and wood forests [8, 9], climate change [10], and the use of these plants as ingredients by the agri-food industry [11, 12], in addition to the loss of traditional background agrobiodiversity knowledge by indigenous people.

One of the most common methods for learning the conservation status, management, and use of edible wild plants is through ethnosemantic and ethnoecological evidence. Both forms of evidence result from ordinary forms of perception of the plant world developed by human groups, and they can be analyzed in every species [13, 14]. From the ethnobotanical point of view, it is well known and documented that indigenous people maintain a close relationship with wild edible plants and value them for their social, economic, and ecological importance [15–18]. It is also necessary to take into account the influence of anthropogenic factors on the state of conservation, presence, and distribution of wild edible species. Thus, it is necessary to revisit some formal investigations which have reported the use of statistical spatial models of plant distribution and have demonstrated its value as a useful tool [19, 20]. The identification of suitable habitats for the proliferation of a particular species could result in the detection of subtle physical changes that could affect their geographical potential distribution area [21], for both wild and cultivated species.

One of the wild, native plants of Mesoamerica widely used as a food ingredient and used in traditional medicine is *Smilax aristolochiifolia* [22–24]. *S. aristolochiifolia* is a perennial woody climber, native to Mexico and Central America, whose actual distribution comprises Costa Rica, Guatemala, Belize, and Mexico [25]. Within the Mexican land territory, *S. aristolochiifolia* is distributed among the states of Chiapas, Campeche, Tabasco, Oaxaca, San Luis Potosí, Tamaulipas [25], Puebla [23], and Veracruz [26].

In the state of Puebla, *S. aristolochiifolia* is distributed along the northern and northwest Sierra of Puebla. In this region, tender stems of the plant are used as a food ingredient in local recipes and are used to treat certain diseases and medical conditions: the indigenous Totonac and Nahua cultures use the plant to treat menstrual pain and dysentery [24]. In this region, *S. aristolochiifolia* is found in tropical forest, acahual (a type of traditional agroecosystem with local secondary vegetation), and coffee plantations, mainly in the municipalities of Tuzamapan de Galeana, Zapotitlán de Méndez, Cuetzalan del Progreso, and Xochitlán de Vicente Suárez [23, 24]. Despite its importance, according to previous reports obtained from four data collections in the study area, the geographical distribution of *S. aristolochiifolia* has significantly declined.

Due to the above, the current research begins with the following research questions:

- What is the perception of the Totonac people about the abundance and distribution of *S. aristolochiifolia* over the last 50 years?
- Based on the knowledge of the Totonac people, what are the main factors that have contributed to the decline of kgentsililh in their communities in the last 50 years?
- What is the potential geographical distribution of *S. aristolochiifolia* in the state of Puebla?
- What are the main physical and climatic factors that determine its geographical distribution?

The first hypothesis is that *S. aristolochiifolia* continues to maintain its food and medicinal value, despite its relatively low abundance and distribution over the last 50 years, according to the perception of the villagers, which is a consequence of changes in land use, herbicide applications, and overexploitation in the Poblano Totonacapan.

The second hypothesis is that the main parameters that affect the distribution of *S. aristolochiifolia* are related to physical climate variables such as altitude and rainfall patterns. This formulation arises because the plant grows in secondary forest vegetation (acahual type agroecosystems), coffee plantations, and old-growth forests (evergreen lowland forest).

## Background information

Smilacaceae are a monocot family lianas, shrubs, and herbs, which are widely distributed in tropical and subtropical ecosystems but also inhabit temperate zones in the Earth’s northern and southern hemispheres [27]. Based on previous floristic and monographic studies, which describe around 350 different species of the Smilacaceae family, Ferrufino-Acosta [25], Li [28], and Govaerts [29] nevertheless argue that at least 40% of the total species described are related simply as synonyms. This means that only 210 species of the Smilacaceae family could be recognized [24]. In Central and South America it is reported that the Smilax genus consists approximately of 260 species (including synonyms) [30], whereas only 28 are endemic species of Mexico [31]. One of these species is *S. aristolochiifolia* Mill., which is a wild species, and is found commonly in the wet tropical forests of Mexico between 100 and 800 m asl [25, 32]. Studies performed in this country reported specific details of the habitat of *S. aristolochiifolia,* and its distribution comprises humid and sub-humid tropical regions from 15 to 800 m asl [25, 26]. By contrast, Ruíz-Sánchez et al. [33] reported a creeper specimen in a coniferous forest between 2200 and 2724 m asl in the State of Durango; according to the authors, this may be considered as a humidity index indicator. The growth of this species in such environmental conditions has been reported in eight Mexican states [23, 25, 26].

## Study area

The Poblano Totonacapan comprises the northern part of the State of Puebla, which has high cultural and biological importance in Mexico [34, 35]. Several authors have reported around 600 useful plant species, used mainly for food, medicinal, and ornamental purposes, but they are also known to have a further 20 different uses [24]. Around 200 plant species are used as food ingredients and employed in indigenous traditional food recipes. These are grown in coffee plantations, family gardens, traditional milpas, acahual agroecosystems, and chili and bean plantations. The local plant biodiversity results from agricultural practices such as the slash and burn method [23, 36–39]. It is well known that the subsistence alimentary strategy of the Totonac communities depends broadly on the utilization of a great diversity of useful plants, including wild, tolerated, and cultivated species [24, 40], among which the corn crop plays a key role [41].

The study took place in 13 locations of the Totonacapan region of Puebla. These localities have previously reported the presence of *S. aristolochiifolia* [24], and we include new sites where local people in cutting communities reported sightings of the plant. All the target sites are distributed in environments with hot and humid A(f) and semi-warm humid (A)C(fm) climates at altitudes from 250 to 1360 m asl (Table 1). The current report uses the terms “Totonac indigenous people”, “Totonac communities”, and “Totonac indigenous populations” to refer to the 13 communities which belong to the Poblano Totonacapan.

**Table 1:** Description of study locations

Community	Municipalities	Altitude (m.a.s.l.)	Climate
Ozelonacaxtla	Huehuetla	850	A(f)
San Rafael	Ixtepec	500	A(f)
Tetelilla	Tuzamapan	540	A(f)
Tzinacapan	Cuetzalan	850	A(f)
Ayotzinapan	Cuetzalan	582	A(f)
Cucuchuchut	Caxhuacan	390	A(f)
Caxhuacan	Caxhuacan	705	A(f)
Zoquiapan	Zoquiapan	1010	(A)C(fm)
Olintla	Olintla	541	A(f)
Atlequizayan	Atlequizayan	830	A(f)
Tuxtla	Zapotitlán de Méndez	820	A(f)
Tapayula	Camocuautla	1200	(A)C(fm)
Cuautotola	Amixtlán	1360	(A)C(fm)

In all localities studied, families were interviewed, of which at least one member was 60 years old or older. An additional consideration was that the family still cultivated maize, and knew or at some point had used the kgentsililh species either as food or for medicinal purposes.

### Ethnobotanical evidence

We conducted 260 surveys among rural indigenous people in 13 different municipalities (Table 1). At the field level, during the collection of specimens of *S. aristolochiifolia,* every target site was georeferenced and recorded. The surveys considered the following questions (variables): consumption frequency, use in traditional medicine, abundance and distribution perception, convincing arguments about the geographical area reduction of specimens, harvesting dates, the cutting community, and local market prices. In addition, every survey contained a further section for collecting traditional gastronomic and medicinal customs related to *S. aristolochiifolia*. Regarding the variable “Abundance and perceived distribution”, the central questions were: “What was the abundance and distribution of the Kgentsilih 50 years ago?” and “What is the current perception about the abundance and distribution of kgentsililh?” The answers were valued using the scale proposed by Blancas et al. [42], according to relative abundance (very low 1; low 2: regular 3; high 4; very large 5) and distribution (restricted 1; wide 2).

### Distribution modeling, abundance, and identification of *S. aristolochiifolia*

The Bioclim and Maxent statistical models enable the estimation of the geographical spatial distribution of wild species such as *Jefea* genus (Asteraceae) [43], *Agave potatorum* [44], *Prosopis flexuosa* [45], *Vanilla planifolia* [46], and *Carica papaya* [47]. However, the Maxent model has so far proved the most reliable for the analysis of the spatial distribution of a particular species because it does not take sampling bias into account. Therefore, it is possible to predict geographical distribution by adding physical parameter data together with specific and supplemented plant data with some predictable continuous and categorical variables [48, 49].

**Fig. 1:**
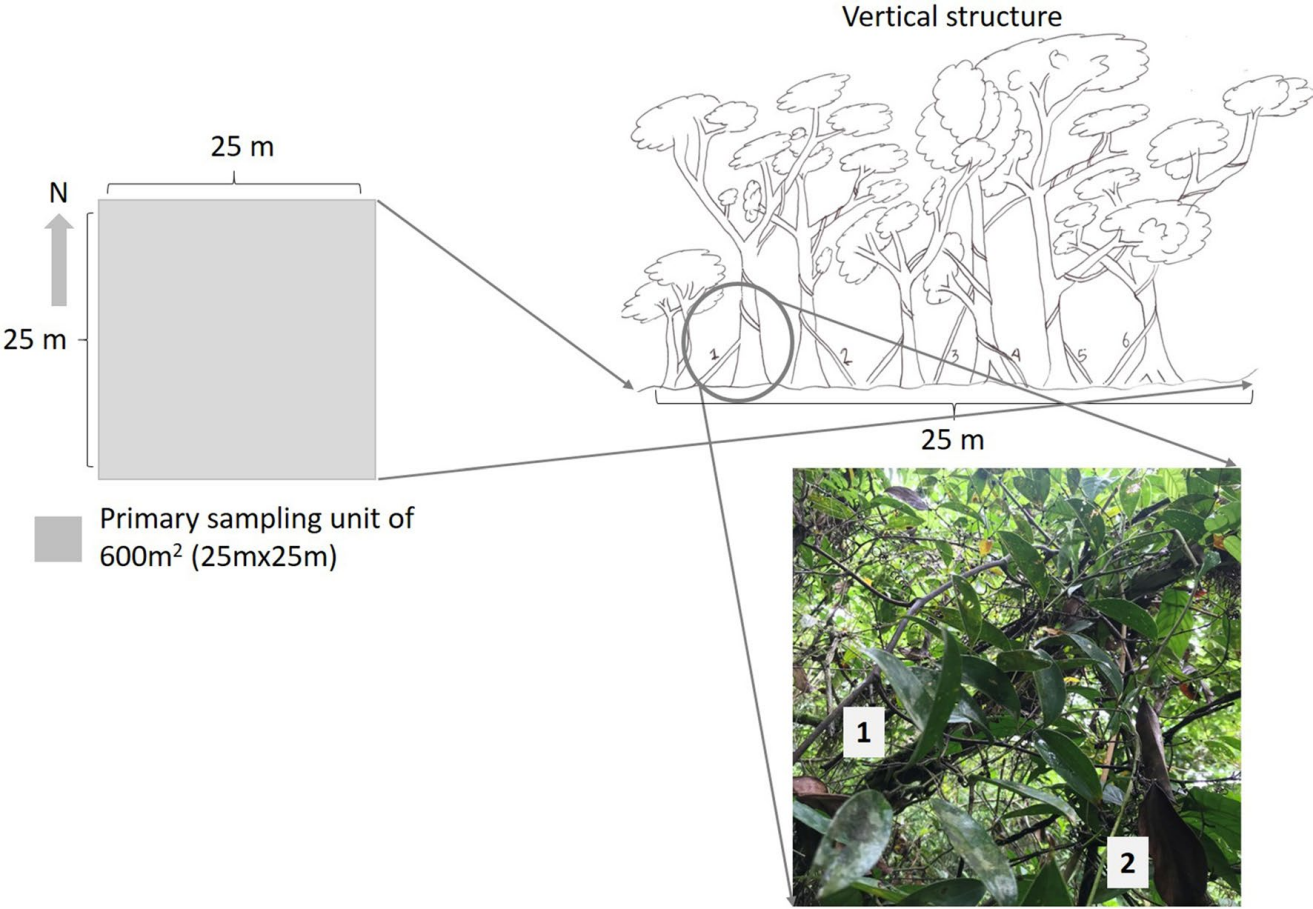
Scheme of the sampling method of *S. aristolochiifolia*

To this end, between 2019 to 2020, the presence of *S. aristolochiifolia* was located in the field using the method known as “walk in the woods” [44, 50] during the collecting months (March, April, and September) and also during the flowering season (November). Although kgentsililh is a perennial plant, during the months of its collection it has a higher abundance of biomass (new plant guides or young shoots). To include individuals of the target species during fieldwork, we set up 25 m × 25 m temporary sampling plots [51] (Fig. 1). This sampling procedure was mainly determined by the growth habit and life form of *S. aristolochiifolia*, which is a scarce prostrate similar to clambering semi-woody vines.

Ultimately, 22 quadrats of 600 m^2^ were established in milpas agroecosystems, coffee plantations, acahual, and old-growth forests (Table 2). Every collection of specimens was georeferenced with the Global Positioning System (GPS Garmin Etrex 32x®). During the examination, 32 individuals (abundance) were identified in situ, distributed among the 22 quadrats.

**Table 2:** Features of sampling sites of *S. aristolochiifolia* on the Totonacapan land territory of Puebla

Agroforestry system	Number of sampling sites	Number of individuals	Abundance (mean)	Density individuals per hectare	% slope steepness (average)
Acahual*	7	13	1.8	31	27
Coffee plantations	6	7	1.1	19	24
Milpa	1	1	1	16	21
Old-growth forest	8	11	1.3	23	33

*Post-cultivation vegetation successions

Later, we estimated the geographical spatial distribution and habitat suitability using Maxent Program Ver. 3.3.3 [52, 53]. To carry out the analysis, we used 22 variables as a predictor (Table 3), of which 19 were bioclimatic variables of high spatial resolution (0.5 arc minutes); this information was downloaded from the WorldClim (worldclim.org) database. Three forms of environmental data were also considered: (1) digital terrain elevation data (DEM; 30 m of resolution), obtained from the geographical elevation continuum of Mexico 3.0 [54], (2) “shapefile” shells of usable land and vegetation in Mexico [55], and (3) the soil moisture regime [56].

**Table 3:** Environmental and bioclimatic variables used to assess the geographical potential distribution area of *S. aristolochiifolia* in the State of Puebla, Mexico

Code	Variable description	Units
Bio1	Average annual temperature	°C
Bio2	Diurnal temperature oscillation	°C
Bio3	Isothermality	Dimensionless
Bio4	Temperature seasonality	CV
Bio5	Average maximum temperature of the warmest period	°C
Bio6	Average minimum temperature of the coldest period	°C
Bio7	Annual temperature oscillation	°C
Bio8	Average temperature of the rainiest four-month period	°C
Bio9	Average temperature of the driest four-month period	°C
Bio10	Average temperature of the warmest quarter	°C
Bio11	Average temperature of the coldest quarter	°C
Bio12	Annual precipitation	mm
Bio13	Precipitation of the rainiest period	mm
Bio14	Precipitation of the driest period	mm
Bio15	Seasonality of precipitation	CV
Bio16	Precipitation of the wettest four-month period	mm
Bio17	Precipitation of the driest four-month period	mm
Bio18	Precipitation of the hottest quarter	mm
Bio19	Coldest quarter precipitation	mm
Bio20	Altitude	m
Bio21	Humidity regime	Days
Bio22	Ground cover	23 types

°C = degree Celsius, CV = coefficient of variation; m = meters; mm = millimeters

### Data analysis and description of potential geographical areas of distribution

Data analysis was performed with non-parametric and parametric tests using the SPSS statistical package V.20.0.0.0. First, the consumption frequency of *S. aristolochiifolia* was converted to a normality test. In addition, a Chi-squared test was performed to distinguish between data abundance perception and associated factors related to the abundance of *S. aristolochiifolia.*

Finally, using the Maxent Program Gradient File, layers of the potential distribution area were obtained based on field data. The layers were exported to ArcMap Program Ver. 10.3 [2018] and were converted to a vector format to estimate the potential geographical area distribution of *S. aristolochiifolia*.

### Origin of the name of the species

**Fig. 2:**
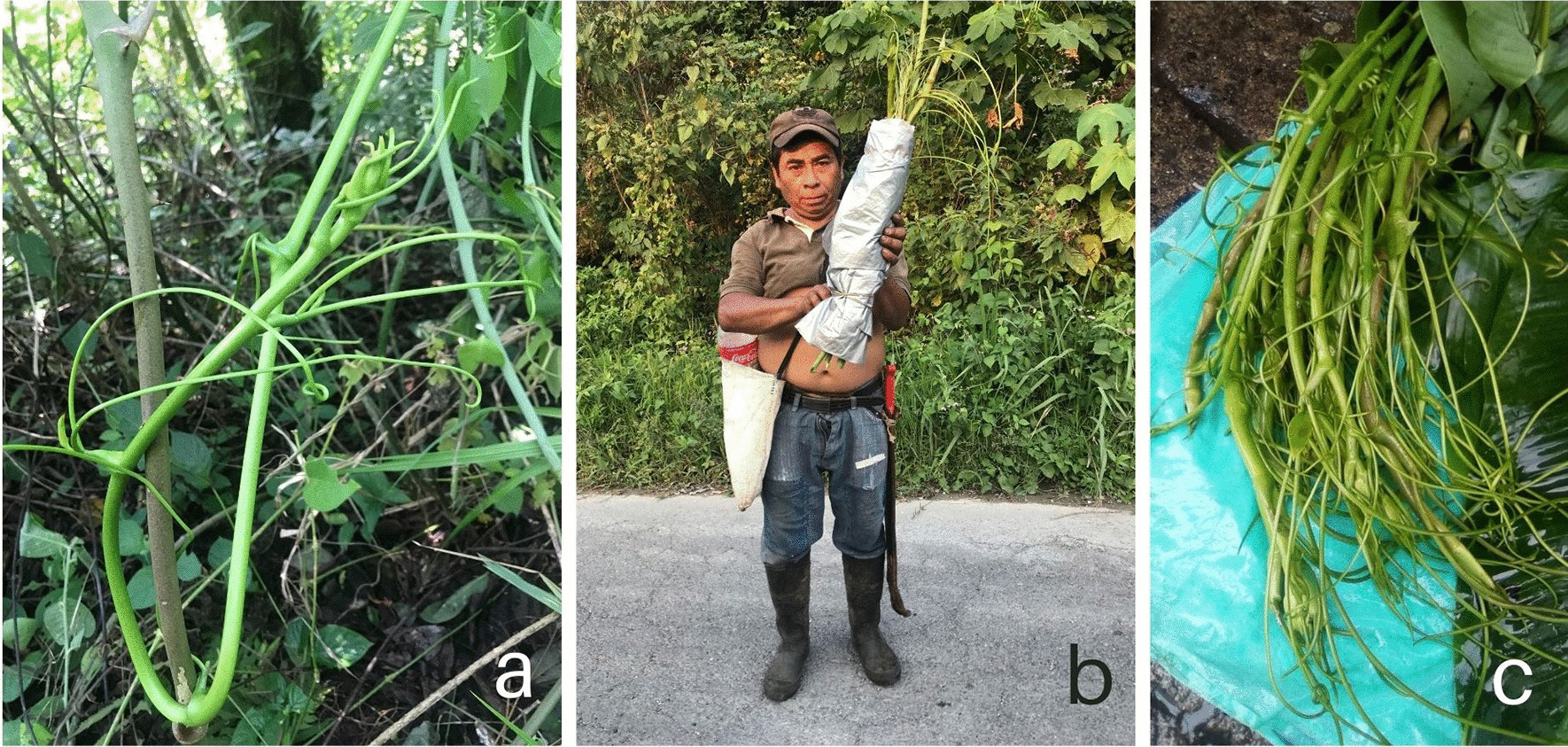
Collection and sale of *S. aristolochiifolia* in the northern Sierra of Puebla

*Smilax aristolochiifolia* is well known as “*kgentsililh*” or “*kgantsililh*”, depending on the variant of the Totonac language. The name kgentsililh is a compound word derived from kgen/kgan and tsililh, and could be translated into English as “dark shrimp nose.” This name comes from the close resemblance of the apical part of the *S. aristolochiifolia* to the antennae of dark shrimp (*Macrobrachium acanthurus*), and therefore (Fig. 2a,c) in Spanish, the species is also known as “bigotes de camalla”, “cosole” or “zarsaparrilla” [57].

### Abundance perception

*Smilax aristolochiifolia* is a species with high socioeconomic importance: some families earn income from the commercialization of its cuttings (Fig. 2b), and it is a significant culinary item. The villagers reported that over the last 50 years, the populations of this plant had diminished in the northern Sierra of the State of Puebla. According to the indigenous Totonac families, changes in land use began in 1970, when the traditional system of food production named acahuals and the old-growth forest were displaced by coffee-type plantations. This phenomenon caused the displacement or elimination of wild plants such kgentsililh. As well as the change in land use, the Totonac people said that factors such as herbicide applications on milpas and coffee plantations, climate change, over-collection, and urban growth had contributed to the population decrease of kgentsililh.

**Fig. 3:**
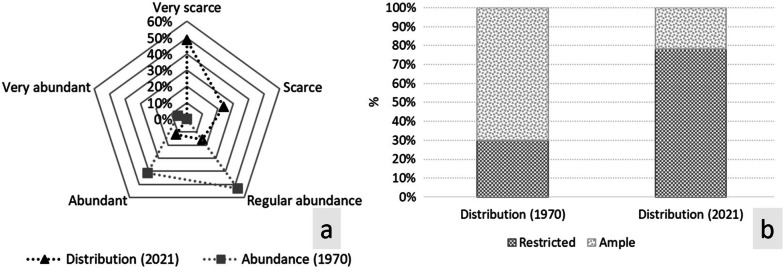
Distribution and abundance perception of *Smilax aristolochiifolia* in two historical times (1970 and 2021)

The Totonac people’s perception about the relative abundance of the species 50 years ago ranged between “regular abundance” (53%) and “abundant” (41%). Regarding distribution, the main perception of the Totonac people was that this had been “wide” (78%). The current abundance and distribution were mainly perceived by Totonac people to be “low” (49%) and “restricted” (78%), respectively (Fig. 3a, b).

These people's perceptions of abundance and distribution, according to the results of the Chi-squared test, reveal no relationship between associated factors in the population decrease of *S*. *aristolochiifolia* (Tables 4, 5).

**Table 4:** Abundance perception concerning the factors associated with the reduction presence of *S. aristolochiifolia* in the northern Sierra of State of Puebla

What factors are associated with the decline in the Kgentsililh population?	What is your perception of the abundance of the species?				Total
	Very scarce	Scarce	Regular abundance	Abundant	
Changes in land use	23	17	10	9	59
Herbicide use	61	31	16	13	121
Climate change	23	10	9	6	48
Over-collection	16	1	5	2	24
Urban growth increase	4	2	1	1	8
Total	127 (49%)	61 (23%)	41 (16%)	31 (12%)	260

Chi^2^ = 9.955, *df* = 12, *p* = 0.620

**Table 5:** Distribution perception concerning the factors associated with the reduction presence of *S. aristolochiifolia* in the northern Sierra of State of Puebla

What factors are associated with the decline in the Kgentsililh population?	What is your perception of the abundance of the species?		Total
	Restricted	Broad	
Changes in land use	48	11	59
Herbicide use	88	33	121
Climate change	41	7	48
Over-collection	18	6	24
Urban growth increase	8	0	8
Total	203	57	260

Chi^2^ = 6.283, *df* = 4, *p* = 0.179

### Nutritional and medicinal importance

**Fig. 4:**
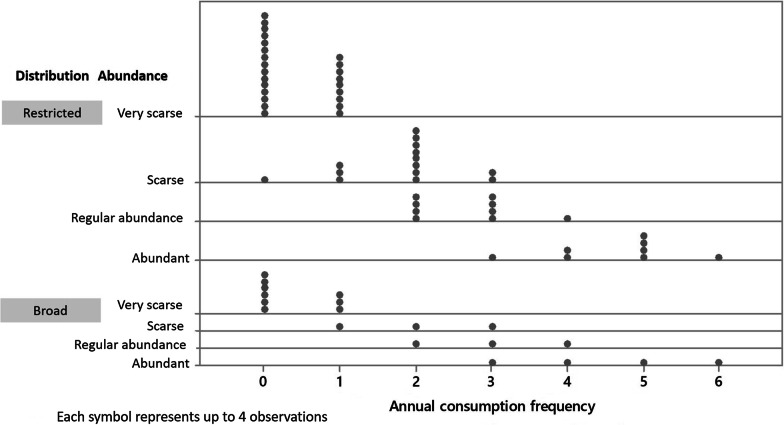
Consumption frequency of *S. aristolochiifolia* in the Totonacapan region of Puebla

As an alimentary issue, this study demonstrated that *S. aristolochiifolia* is consumed by the indigenous Totonac people throughout most of the year. 82 of the families interviewed said that they had not included kgentsililh in their diet or had stopped eating it. The consumption frequency is concentrated once or twice each year (Fig. 4). It is important to recall that some of the Totonac people consider the species to have restricted distribution and low abundance and these are the same people who do not consume kgentsililh as a food ingredient nor use it in traditional medicine. As the frequency of consumption increases, there is a perception that the plant has “regular abundance” and “very large abundance” (Fig. 4).

**Fig. 5:**
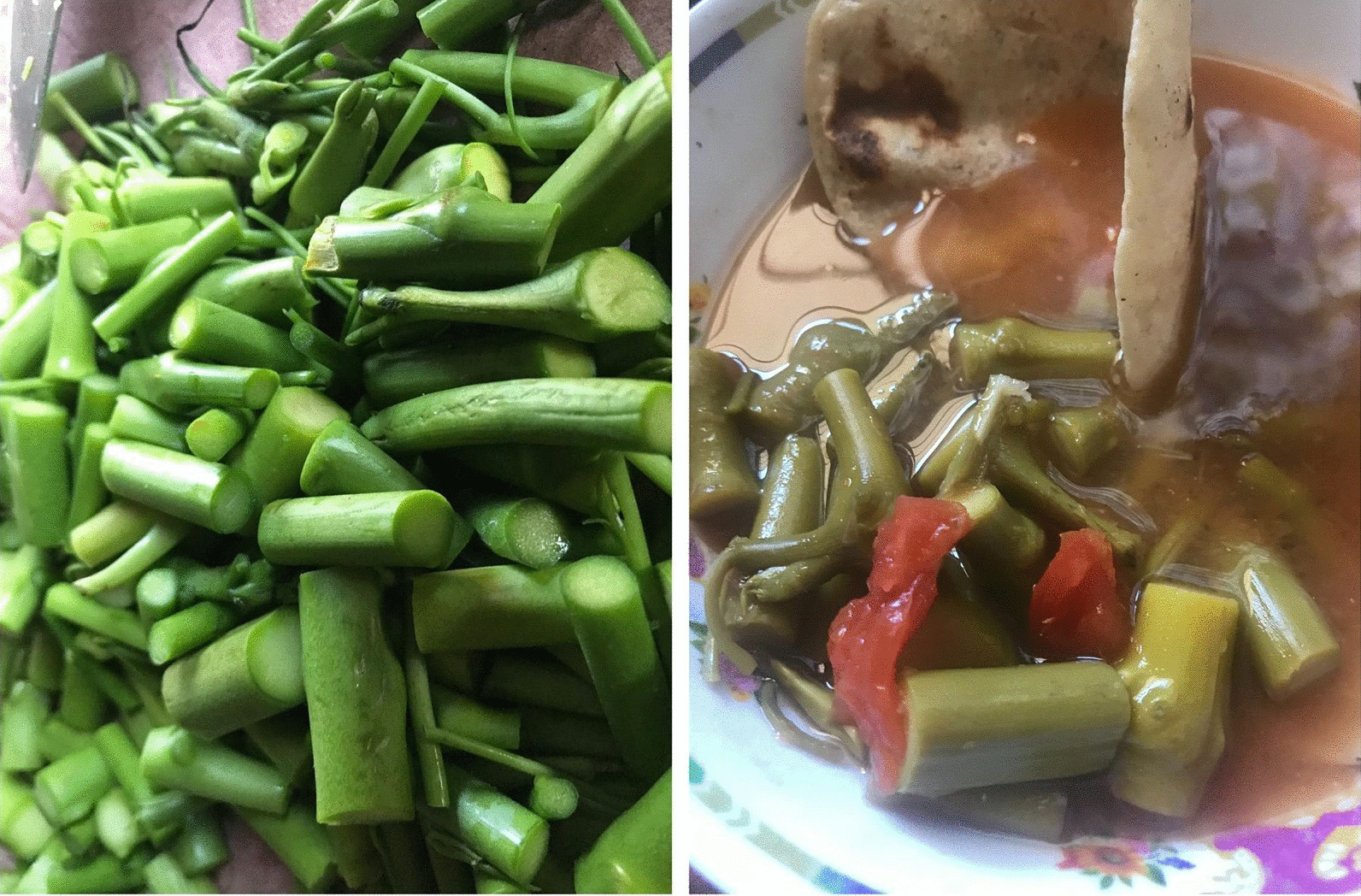
Totonac recipes with *Smilax aristolochiifolia*

The Totonac indigenous families argued that the main reasons for the low rate of consumption of *S. aristolochiifolia* could be the following factors: (a) low availability of the plant, (b) changes in the dietary patterns of new generations, and (c) lack of knowledge of local culinary recipes. We found three different recipes for preparing *S. aristolochiifolia* as a food source: (a) “Enchiladas”, which consist of the plant guide cut into slices of approximately 5 cm and boiled with epazote leaves and pieces of serrano chili or chiltepin chili (Fig. 5); (b) as an ingredient, cooking the guide in boiling water with the addition of corn dough and beans; and (c) Chilpozo, which is prepared by adding slices of *S. aristolochiifoli*a to boiled dark shrimp.

In traditional medicine, according to the findings of this investigation, the Totonac people reported that *S. aristolochiifolia* is used to treat baldness and stomach pain. In the first case, a tender guide plant is cut, and the sap is extracted and spread over the head; this process is used for women who have given birth and for children whose hair cannot grow. For the second treatment, an extract called “tuberous root” is obtained from the rhizome of *S. aristolochiifolia,* and it is then washed and dried to prepare a tea infusion. On average, around 100 g of crushed “tuberous root” of *S. aristolochiifolia* are used with a liter of boiled water.

### Economic importance

Concerning economic issues, the average cost of plant bundles varies from 10.00 to 40.00 Mexican pesos (around 0.5 to 2.00 US dollars), and every bundle consists of between seven and 10 cuttings sized around 0.4 m to 0.5 m (Fig. 2b). The cost of the plant bundles depends on whether they are sold in the same community (inside local traditional markets) or in regional markets. If marketing takes place inside the community, this implies no transportation or food cost for the collector, and therefore every bundle costs no more than 10.00 Mexican pesos. However, if kgentsililh is marketed in regional markets, which requires transportation to the municipal seats, each bundle can cost between 30.00 and 40.00 Mexican pesos. In all communities, there are five to seven people engaged in the collection and commercialization of the species. In general, their income is between 120.00 and 470.00 Mexican pesos per year (5.5 to 21.4 US dollars).

The Totonac people dedicated to the collection of kgentsililh are men, women, and children from the community. As well as collecting this plant, they save other plants and fruits such as *Diospyros nigra, Pouteria sapota, Licania platypus*, *Persea schiedeana*, *Spondias mombin,* and quelites. However, the people who buy Kgensilih and other products from these collectors are those who no longer dedicate themselves full-time to fieldwork, but have still maintained a culinary preference for this type of food.

### Local management

In 22 quadrats, 32 *S. Aristolochiifolia* individuals were identified. Acahuals and old-growth forest provided the largest numbers, at 13 and 11 individuals, respectively. The numbers for the four sample sites show an estimated average density per hectare of 31 specimens in acahual, 23 in old-growth forest, 19 in coffee-type plantations, and 16 in milpas (Table 2). During March, April, July, August, September, and October, which correspond to the periods of the highest abundance of plant structure, “young shoots or new plant guides” are particularly consumed (Fig. 1b). *S. aristolochiifolia* is only collected in acahuals and old-growth forests, and the plant, therefore, does not receive management or care. Kgentsililh is a tolerated plant in coffee-type plantations and milpas, where its management consists basically of the removal of potential competitors. Unfortunately, there is no special interest in increasing the number of plants, nor is there an artificial selection of the species in any of the places where the plant is located.

### Potential distribution of *S. aristolochiifolia*

Eight of the 22 variables (Table 6) used for an abundance predictor model of the potential distribution of *S. aristolochiifolia* contributed significantly to the understanding of the most suitable habitat distribution of kgentsililh, among them, precipitation in the driest month (51.7%), the warmest quarter (38.3%), and ground cover (3.7%). Other variables completed the distribution model, such as humidity regime (2.2%), altitude (1.8%), annual precipitation (1.4%), the average temperature in the driest four-month period (0.9%), and rainfall in the driest four-month period (0.1%).

**Table 6:** Percentage contribution of bioclimatic variables in prediction distribution models for *S. aristolochiifolia* on the State of Puebla, Mexico

Variable	Percent contribution (%)
Precipitation of the driest period (Bio14)	51.7
Precipitation of the warmest quarter(Bio18)	38.3
Ground cover (Bio22)	3.7
Humidity regime (Bio21)	2.2
Altitude (Bio20)	1.8
Annual precipitation (Bio12)	1.4
Average temperature of the driest four-month period (Bio9)	0.9
Precipitation of the driest four-month period (Bio17)	0.1

**Fig. 6:**
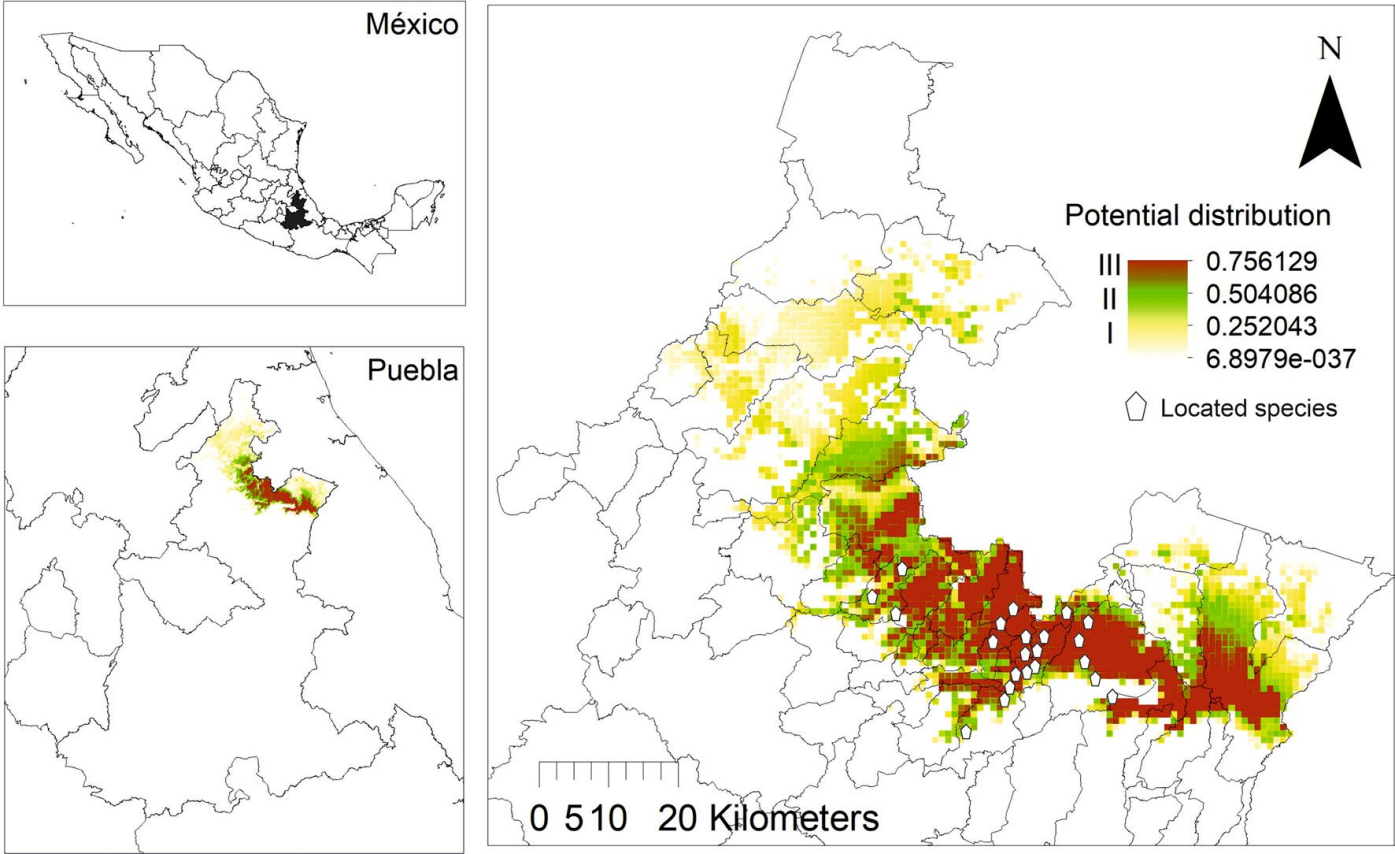
Potential geographical distribution of *S. aristolochiifolia* in the State of Puebla

According to our results, the potential land area distribution of *S. aristolochiifolia* covers 37 municipalities of the State of Puebla, 11 of which correspond to this study. The analysis is divided into three areas. Potential Area I (gray yellow) is characterized by a habitat of low quality (Fig. 4); this area covers 430.2 km^2^, distributed among 24 municipalities. Potential Area II, marked in green, covers 548.5 km^2^ and comprises 18 communities. The most suitable habitat for the growth of kgentsililh is Potential Area III, which covers 574.9 km^2^ and comprises 21 communities (Fig. 6).

In 37 municipalities environmental suitability was considered, whereby the mathematical or statistical relationships between the known distribution and a set of independent variables were used as indicators for the favorable development of the species *S. aristolochiifolia*. With this information, programs can be developed for the conservation or sustainable use of kgentsililh. There are some potential problems with this species due to over-collection, growth of the urban areas, and lack of interest in improving its conservation through clonal reproduction or artificial selection. To date, the presence of the species has not been corroborated in the potential zones of distribution, since we do not have a methodological design to verify the success of our proposal.

Area III corresponds to the highest habitat quality for the species and presents the following features: semi-warm humid climate of group C, average annual temperature over 18 °C, and less than 18 °C in the coldest month, and altitude ranging from 285 to 950 m asl. In addition, this area has an annual rainfall of between 2500 and 4000 mm, the warmer quarter varying between 800 and 1500 mm, and a udic moisture regime level from 330 to 365 days. The characteristic vegetation varies from medium sub-deciduous forest to high evergreen forest. It is important to recall that *S. aristolochiifolia* depends widely on having little disturbance of natural vegetation and friendly farming practices for its survival; therefore, the species grows on coffee and acahual plantations and milpa farms.

On the other hand, Area I and Area II have low-to-medium habitat qualities and are well characterized by their warm-humid climate, with an average annual temperature greater than 22 °C and a coldest-month temperature greater than 18 °C, altitude lower than 200 m asl, and a udic moisture regime between 270 and 330 days. The areas are characterized by their deciduous forest vegetation, and their land is used mainly for monoculture plantations of citrus, banana trees, corn, and apple orchards.

### Use of *S. aristolochiifolia* in food and medicine

**Fig. 7:**
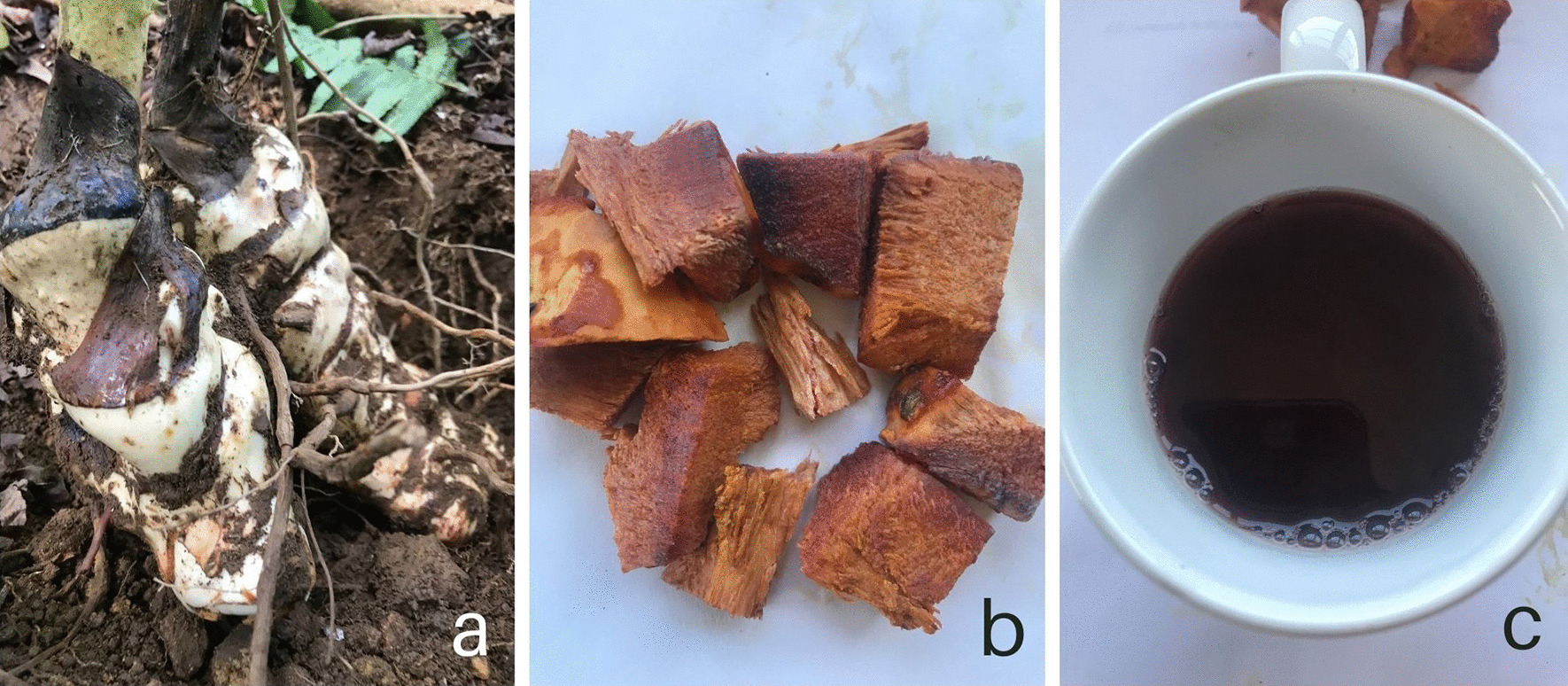
Procedure for oral infusion preparation of *S. aristolochiifolia.* **a** “Tuberous root” extraction, **b** trituration, washing, and drying of “tuberous root” (**c**), tea infusion preparation

*Smilax aristolochiifolia* is employed as a food ingredient in around 68% of family homes interviewed, independently of the frequency of consumption. In the northern Sierra of the State of Puebla, the tender parts of the stems have historically been prepared according to local indigenous recipes such as roasting, boiling, stewing, or submersion in vinegar like asparagus [23, 24] (Fig. 7). Without a doubt, the low consumption frequency of *S. aristolochiifolia* by the indigenous people is due to the following factors: adoption of new eating patterns by the new generations, the relatively low abundance of species, and lack of knowledge of traditional recipes. In contemporary Mexico, as in the global context, the agri-food industry has modified consumer preferences [11, 12], including those of the indigenous population [58], and this is reflected in the change of ingredients in traditional culinary recipes.

In Mexican traditional medicine, the part of *S. aristolochiifolia* most frequently consumed as “tuberous root” is obtained from the rhizome and the sap contained in the stalks. All people interviewed use the tender stems and “tuberous root” to treat baldness and stomach pain, although the study area has also reported the use of the plant to treat menstrual pain and dysentery [24, 59]. In the state of Queretaro, in Mexico, the Otomies and Nahuas cultures use the plant to prepare an oral infusion from “tuberous root” to treat obesity [59–61]. In the Chinanteca culture of the state of Oaxaca, Mexico, the people prepare a tea infusion from “tuberous root” to deal with a dermatological disease known as “mal del pinto” [62]. In other regions of Mexico, a tea infusion is prepared from the “tuberous root” of *S. aristolochiifolia,* and is consumed as a blood purifier and as a treatment against syphilis, diabetes, stomach infections, and seasonal influenza [63, 64]. According to previous ethnobotanical reports, *S. aristolochiifolia* is not only used in traditional medicine in rural areas in Mexico, including indigenous land areas but also in certain urban areas of foreign countries [65]. The plant is used in the cosmetic industry to manufacture female aphrodisiac products, which are marketed in foreign countries such as the USA [66].

Additionally, *S. aristolochiifolia* is a forest biological resource of extreme importance; for example, the Totonac and Huasteca cultures use the dried stems of the plant to create basket back ribs and traps in the form of packing cases to catch fish [24, 67]. At the same time, the stems and leaves of *S. aristolochiifolia* provide ingredients for the brewing, soft-drink, and confectionery industries. The roots are the source of ingredients for aromatizing beers, due to their characteristic bitter and sweet flavors [68].

### Factors associated with the decline of the *S. aristolochiifolia* population

The perception of the inhabitants concerning the abundance and distribution of *S. aristolochiifolia* is low compared to the panorama of 50 years ago. According to the indigenous people, this is due mainly to land-use changes and the application of herbicides in traditional production areas. In the northern Sierra of Puebla, since the 1960s, coffee plantations have expanded as a monoculture, with shade and secondary vegetation types gradually replacing 41.9% of the old-growth forest [69] and therefore exacerbating the elimination of wild plants such as *S. aristolochiifolia.* It is important to emphasize that the highest abundance of the plant is in acahual agroecosystems (Table 2). Originally, these cultivations served as spaces for pasture and agriculture. Over time, these spaces became coffee plantations with intermediate secondary vegetation of tropical forest or mountain mesophyll forest [69].

In addition, the application of herbicides mainly in the milpa agroecosystems directly impacts the presence or absence of some characteristic plants such as quelite [70] and kgentsililh. The use of milpa agroecosystems in the North Sierra of Puebla for subsistence agriculture, the use of chemical fertilizers, and the occasional application of herbicides to control weeds could be influencing the low abundance of *S. aristolochiifolia* in such unit production (Table 2). The first effect of a change of land use for agricultural and cattle production is a significant reduction in the semi-wood vine population, which in some places have almost reached the point of disappearance (Douma et al. 2016).

### The utility of prediction models

The potential distribution and relative abundance of agriculturally important and priority species can be addressed by bioclimatic models, ecological niche models (ENM), and species distribution models (SDM) [71–73]. Among these is the program MaxEnt [74], which models the geographic distribution of species with presence records and determines their arrangement through the probability of maximum entropy bounded by a set of known covariates such as climate, topography, soils, and vegetation [75]. In our study area, *S. aristolochiifolia* grows in tropical forests, acahual, and coffee plantations [23]. It is therefore considered that an initial potential model can be elaborated with the individuals located and distributed in the 13 localities. The MaxEnt program performs a relative estimation to determine the contribution of every variable in the modeling, and adds an increase of regularized gain to the corresponding variable or subtracts from it if the change of absolute value is negative [76]. In this way, contributions are assigned to the environmental variables on which every species depends, and finally, the values are converted into percentages to obtain a contribution table.

Currently, the National Information System on Biodiversity (NISB) in Mexico reports 157 collections of *S. aristolochiifolia,* five of them made by naturalists. In the literature, the State of Puebla has described four collections. The first was carried out in the Venustiano Carranza municipality in 1960, the second in the Tuzamapan de Galeana municipality in 1980, and the third in the Cuetzalan del Progreso municipality in 1998 [57]. The last was carried out by Martínez-Alfaro et al. [24] in Zapotitlán de Méndez and Xochitlán de Vicente Suárez. This means that the presence of kgentsililh has previously been reported in five municipalities in the northern Sierra of Puebla. In this current research, we were able to georeference its growth in 22 locations and collect 32 specimens of *S.* a*ristolochiifolia* distributed in 13 localities. Moreover, the distribution model allows the identification of 14 other localities with a potential presence of kgentsililh, and the distribution of this plant could include almost 25 municipalities which have a total area of 303,712.2 hectares and are a suitable habitat for *S. aristolochiifolia* (Additional file 1).

The last point suggests the possibility of finding populations of kgentsililh in the state of Puebla, Mexico: according to the distribution model, these could be from one to four individuals (Annex). These analyses can also be used in further biogeographic, ecological, taxonomic, conservation, and sustainable development studies for *S. aristolochiifolia*. In this respect, knowledge about the potential distribution of kgentsililh could establish the basis for the further field specimen collections of wild populations, which are the central source of genetic background information for improving the nutritional status and yields of cash crops [77]. In addition, this methodology of potential geographical distribution allows the identification of sensitive land areas exposed to future climate change [78]. According to the local indigenous people interviewed in the current study, climate change is one of the main factors that caused the relatively low abundance of *S. aristolochiifolia*, enhancing the importance of knowing and identifying suitable habitats to promote in situ conservation of kgentsililh [20].

In some regions of Mexico, such as Queretaro, the authorities argue that *S. aristolochiifolia* is a species without immediate danger of extinction, but this depends on its presence in the forest and jungle [79]. In our region of study, the soil has changed in use and 41.9% of natural vegetation has been replaced by monocultures and pastures by 2003 [69]. In addition, the increase of urban areas due to population growth [80] has considerably increased the vulnerability of kgentsililh. Finally, in the Northern Sierra of the State of Puebla, several studies have demonstrated the conversion of shrubby old-growth forest to secondary arboreal vegetation over the last 15 years [81]. Nevertheless, the recovery of forest mass is not a good early indicator for the recovery of wild plant populations, especially to herbs and semi-woody prostrate to clambering plants such as kgentsililh [82].

## Conclusions

*Smilax aristolochiifolia* is still a plant of socioeconomic importance, mainly because of its food value and its use in traditional medicine by indigenous families in Poblano Totonacapan. It is evident that the villagers perceive that over the last 50 years the species population has decreased mainly due to land-use change, the application of herbicides to the different family production units, and climate change. At the moment, there is no knowledge about the methods of propagation of the species. However, it would be of great importance to generate a biocultural conservation strategy and take advantage of the results obtained from the potential geographic distribution area, since according to the Maxent® Program, there are still potential areas with suitable habitats to promote conservation in Poblano Totonacapan.

## Supplementary Information

**Additional file 1.** The distribution of Smilax aristolochiifolia individuals in the study area.

## Data Availability

Contact directly with the first author.
